# Spectrophotometric and molecular modelling studies on *in vitro* interaction of tyrosine kinase inhibitor linifanib with bovine serum albumin

**DOI:** 10.1371/journal.pone.0176015

**Published:** 2017-04-18

**Authors:** Tanveer A. Wani, Ahmed H. Bakheit, Seema Zargar, Mohammed A. Hamidaddin, Ibrahim A. Darwish

**Affiliations:** 1 Department of Pharmaceutical Chemistry, College of Pharmacy, King Saud University, Riyadh, Saudi Arabia; 2 Department of Chemistry, Faculty of Science and Technology, Al-Neelain University, Khartoum, Sudan; 3 Department of Biochemistry, College of Science, King Saud University, Riyadh, Saudi Arabia; Islamic Azad University Mashhad Branch, ISLAMIC REPUBLIC OF IRAN

## Abstract

Linifanib (LNF) possess antitumor activity and acts by inhibiting receptor tyrosine kinase VEGF and PDGF. The interaction of BSA with the drug can provide valuable information regarding the pharmacokinetic and pharmacodynamics behavior of drug. In our study the spectrophotometric methods and molecular docking studies were executed to understand the interaction behavior of BSA and LNF. BSA has an intrinsic fluorescence and that fluorescence was quenched by LNF. This quenching process was studied at three different temperatures of 288, 300and 308 K. The interaction between LNF and BSA was due to static quenching because the Ksv (Stern-Volmer constant) at 288 K was higher than at 300 and 308 K. Kq (quenching rate constant) behaved in a similar fashion as the Ksv. Several other parameters like binding constants, number of binding sites and binding energy in addition to molecular docking studies were also used to evaluate the interaction process. A decrease in the binding constants was observed with increasing temperatures and the binding site number approximated unity. The decreasing binding constant indicates LNF–BSA complex stability. The site mark competition experiment confirmed the binding site for LNF was located on site II of BSA. UV–visible studies along with synchronous fluorescence confirm a small change in the conformation of BSA upon interaction with LNF. The thermodynamic analysis provided the values for free energy ΔG^0^, ΔH^0^ and ΔS^0^. The ΔG^0^ at the 288, 300 and 308 K ranged in between -21.5 to -23.3 kJ mol-1, whereas the calculated values of ΔH (-55.91 kJ mol^-1^) and ΔS^0^ (-111.74 J mol^-1^·K^-1^). The experimental and molecular docking results suggest that the interaction between LNF and BSA was spontaneous and they exhibited hydrogen bonding and van der Waals force between them.

## Introduction

Once the drug molecule reaches the systemic circulation it interacts with several biomolecules (predominantly proteins) and this interaction with proteins play a vital role in the pharmacokinetics (absorption, distribution, metabolism and excretion) of drugs [1,2]. Hence studying such interactions is important during the drug discovery and development [3–11]. Predominantly the serum albumin interacts with drugs in systemic circulation, thus studying these binding interactions provides a greater insight into the drug therapy. The binding interaction also provide a information regarding drug–drug interaction and resistance between the drug and protein [12]. Possible drug side effects and dosages can also be predicted using these binding techiniques [13,14]. The pharmacokinetic parameters of drugs like free drug plasma concentration, elimination of drug from body and the distribution of drug in the body depends on the strength with which the drug is bound to serum albumin. BSA was used to study the pharmacological interactions with drugs moieties instead of human serum albumin (HSA) owing to its structural similarity with BSA [15]. In addition to albumin the drug ligands also bind to other proteins such as human holo-transferrin. These proteins act as carriers for these drugs to the site of action [16, 17].

It has been established that inhibition of both PDGFR (Platelet derived growth factor receptor) and VEGFR (Vascular endothelial) receptors together (which play a vital role in tumor cell proliferation and angiogenesis) produce higher antitumor activity than inhibition of either of the receptors alone [18, 19]. LNF also known as ABT-869 is a potent and orally active inhibitor of receptor tyrosine kinases. It inhibits both PDGFR and VEGFR (VEGFR-1, VEGFR-2, PDGFRb, CSF1R) and shows minimal interference with other unrelated receptor tyrosine kinases [20–22]. LNF is currently under investigation in numerous clinical trials and has shown some anti-tumor activity in some cancers. [23–26] LNF is rapidly absorbed post oral administration from systemic circulation with peak plasma concentration of approximately 2 h [27, 28].

Several pharmacokinetic studies for LNF have been performed but amongst them none studied the interaction between LNF and BSA. In the transportation and storage of drug moieties serum albumin plays a critical thus, the study of the biophysical interactions involved will further help in development of LNF molecule [1,2]. The level at which the drug bind to the protein determines its distribution volume and rate of elimination form the body.

These interaction studies therefore, provide a valuable evidence about the structural features and therapeutic efficacy of the drug [29–31]. In this study the LNF and BSA interaction was studied by a combination of experimental and computational approach. These approaches included parameters like quenching constants, binding constants, thermodynamic parameters in addition molecular docking studies. This study is anticipated to give a significant insight to further elucidate the in-vivo storage and transport mechanism of LNF and its pharmacokinetics.

## Materials and methods

### Experimental

#### Reagents

BSA and Diazepam were procured from Sigma Aldrich. LNF and Warfarin were purchased from Weihua Pharma, PRC. Type-IV water was used for buffer preparation.

#### Apparatus

JASCO spectrofluorometric Model FP-8200 and UV–vis spectrophotometer Shimadzu UV-1800 were used for the spectrofluorometric and spectrophotometric experiments. The other instruments used were ELGA water purification system from Elga Lab Water UK.

#### Sample preparation

Stock of LNF was prepared from 5 mg of LNF in 5 mL of acetonitrile with final concentration of 1 mg ml^-1^ whereas, the working standard solution of 2.6 × 10^−4^ M solution was attained by diluting 10 ml of this stock solution with PBS pH 7.4 to 100 mL in volumetric flask. BSA stock was prepared by adding 5 mg of BSA in 5 mL PBS pH 7.4 to get a BSA solution of 1 mg ml^-1^ concentration and a 1.5 μM BSA working solution was obtained from the stock solution by diluting 10 ml of it with PBS pH 7.4 to 100 mL in volumetric flask. These resulting standards were stored in refrigerator (2–8°C).

#### Interactions between LNF–protein

The spectra for fluorescence emission 300–500 nm range with an excitation wavelength of 280 nm was measured. The spectra were recorded at three different temperatures of 288, 300 and 308 K respectively. Several different concentrations of LNF were prepared from the working standard and these concentrations ranged between (0–6.4 µM). to a fixed concentration of BSA (1.5 µM) was added several different concentrations of LNF.

#### Inner filter effect and measurement of synchronous fluorescence

Since, there is a possibility of inner filter effect due absorption by some compounds in the ultraviolet region of excitation or emission wavelength and thus, reduce the fluorescence intensity (FI) [9, 32]. The following equation was used to correct the inner filter effect:

$$\mathrm{Fcor}=\mathrm{Fobs}\times e^{(\mathrm{Aex}+\mathrm{Aem})/2}$$

Where, F_*cor*_ and F_*obs*_ are the corrected and observed fluorescence respectively. A^*ex*^ and A^*em*^ represent sum of LNF absorbance at the excitation and emission wavelengths respectively.

The spectra for the BSA-LNF complex (room temperature) at different intervals of scanning Δλ were acquired. The Δλ values were set to 15nm and 60 nm which characterize the properties of tyrosine and tryptophan respectively.

#### UV–visible spectrophotometric studies

The UV-Visible absorption spectra were acquired for the BSA-LNF complex and LNF alone. To obtain the spectra of the complex, the BSA concentration was held constant (1.5 µM) and LNF concentrations varied from 0 to 6.4 µM (0.0, 1.6, 2.4, 3.2, 4.8 and 6.4). The LNF solution was added to both the BSA as well as the reference solutions so that absorbance due to LNF is eliminated. The samples were studied in the range of 200–400 nm and the spectra recorded.

### Molecular docking

The docking software (Molecular Operating Environment-2014) was utilized for docking experiments between BSA and LNF. The LNF structure was drawn in the MOE software itself. The crystalline structure of BSA with PDB ID (4OR0) was taken from (Protein Data Bank) (http://www.rcsb.org). The minimization of the obtained structures was done by MMFF94x force-field reaction-field with electrostatics (Din = 1, Dout = 80). A flat bottom tether (10.0 kcal/ mol, 0.25 A˚) was also used and applied to all atoms. All modifications were performed in MOE. Root-Mean-Square Deviation (RMSD) values were used to select the best suitable interaction between the ligand and target.

## Results and discussion

### Fluorescence studies

**Fig 1:**
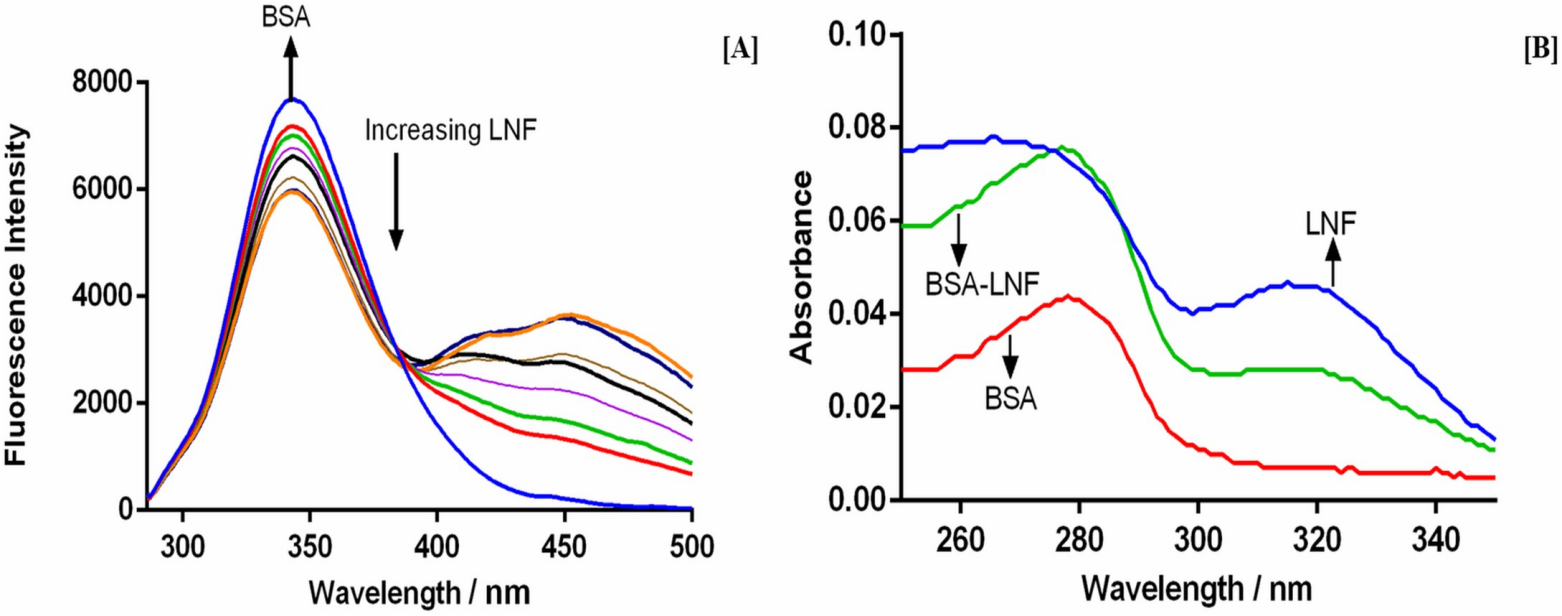
[A] The fluorescence quenching spectra of BSA in presence of different concentration of LNF; [B] UV–vis spectra of BSA, LNF and BSA–LNF complex.

Two amino acid tryptophan residues (Trp-134 and Trp-212) of the BSA molecule are responsible for its fluorescence [33]. The tyrosine residues are also fluorescent but the emission by these residues at the selected excitation wavelength is weak. The positioning of Trp-212 and Trp-134 is within the hydrophobic region of the sub-domain IIA and the surface of the BSA molecule respectively. This positioning of the Trp-134 makes it more susceptible for reactions compared to Trp-212. The excitation wave length of 280 nm (Fig 1A) was used to measure the fluorescence spectra. The analytical samples were prepared with constant BSA concentration and increasingly different LNF concentration. As is evident from the Fig 1A, the intensity of fluorescence of BSA decreased with increasing LNF concentration without any significant emission wavelength shift or change in peak shape demonstrating quenching of intrinsic fluorescence of BSA by LNF. Also an isobestic point is observed at 387 nm indicating the formation of a drug protein complex. The presence of the isobestic point also indicates that an equilibrium is formed between the LNF and BSA and development of 1:1 complex between LNF and BSA.

#### Mechanism of quenching

There are two different possible mechanisms for the quenching process viz, static quenching and dynamic quenching [34]. The difference between the static and dynamic quenching is that a complex formation amongst the quencher and the fluorophore occurs in static quenching where as in dynamic quenching the interaction among quencher and fluorophore is during the lifespan of excited state. The dependence on temperature, viscosity and lifespan measurement differentiates the static quenching from dynamic quenching. With increasing temperature the diffusion and collisional quenching increases and an opposite outcome will occur for the static quenching. The static and dynamic quenching mechanisms can be analyzed with help of Stern-Volmer equation [34].

$$\frac{F}{F_{0}}=1+K_{sv}\left[Q\right]=1+K_{q}\;\tau_{0}\;[Q]$$

**Fig 2:**
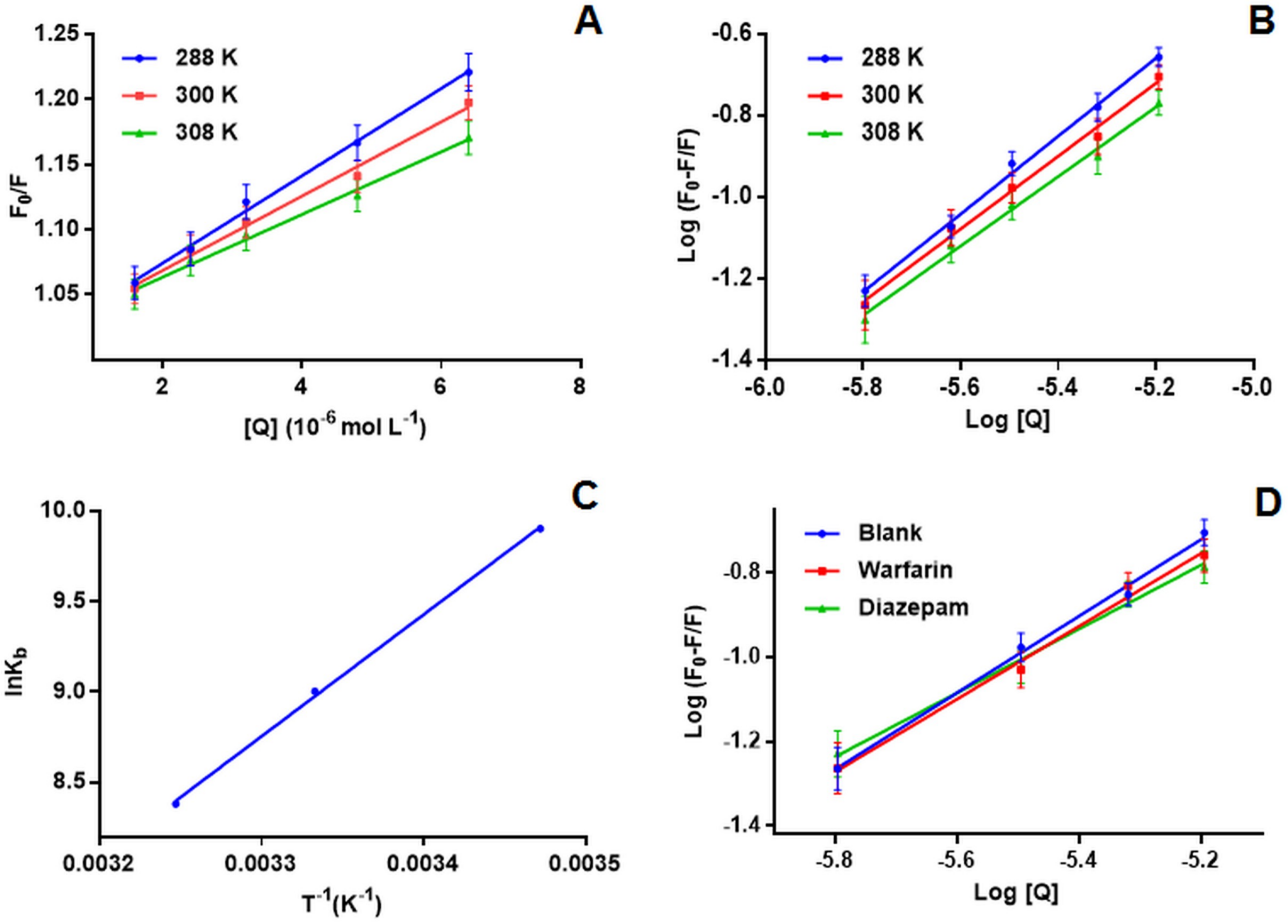
[A] Stern–Volmer plots of F_0_/F vs concentration of LNF; [B] log [(F0‒F)/F] vs log [Q] plot at different temperatures for determination of binding constant; [C] Vant Hoff plot for the BSA with LNF interaction; [D] [(F0‒F)/F] vs log [Q] plot in the presence of warfarin and diazepam.

F_0_ represent the FI in presence of drug; F represent the FI in absence of the drug; Stern-Volmer quenching constant is represented by Ksv, Q is the drug concentration. Kq is the quenching rate constant of biomolecule and τ_0_ is the average lifespan of molecule in absence of quencher and is valued 10^−8^ s. A plot between [Q] (molar concentration) and F_0_/F is linear and the slope equals the Ksv. The obtained results for the Ksv (Stern-Volmer constant) and Kq (quenching rate constant) are present in Table 1 and Fig 2A. Since the Ksv and Kq at 27°C and 35°C were lower the than those at 15°C indicating that the LNF and BSA underwent a static quenching process. Where as in case, there was dynamic quenching an increased binding constant value would have resulted with increased temperature because of increased diffusion. Also, the biopolymers exhibit a maximum of 2.0 × 10^10^ L mol^-1^ s^-1^ diffusion collision quenching rate constant but the binding constants obtained are much higher than this value again specifying the development of a non-fluorescent complex between LNF and BSA (static quenching) [35].

**Table 1:** The quenching constant (K_SV_) and bimolecular quenching rate constant (Kq) for LNF_BSA complex.

T(K)	R	Ksv × 10^−4^ (L mol^-1^)	Kq× 10^−12^ (L mol^-1^s^-1^)
288	0.9958	3.40	3.40
300	0.9918	2.85	2.85
308	0.9928	2.40	2.40

#### Measurement of binding constant and binding site number

The binding constant and the number of binding sites are determined by the modified Stern–Volmer equation (double logarithm regression curve) in which it is presumed that drug molecule binds independently to macromolecule (equivalent sites) [36]

$$\log\frac{\left(F_{0}-F\right)}{F}=\log\;K_{b}+n\;\log[Q]$$

K_b_ represent the binding constant and n is the number of binding sites. The intercept and the slope represent the values of K_b_ and n respectively Table 2 and Fig 2B. The binding constants of BSA-LNF complex at three different temperatures of (288, 300 and 308K) ranged between 2.0 x 104–4.3 x 10^4^ L mol^-1^.The high binding constant values show stong binding interaction between LNF and BSA inferring that the drug will be highly bound in blood plasma in*vivo*. Further, results show a lowering of binding site number at higher temperatures, which could because at higher temeratures the molecules are more disordered, vibrating fast and there is increase in diffusion coefficient which inturn could make the LNF–BSA complex unstable. The value for binding site number is ≈ equal to 1, as no fractional binding sites occur nor there can be <1 binding site, thus inferring binding sites of a single independent class for LNF.

**Table 2:** Binding parameters and thermodynamic parameters of binding process between LNF and BSA.

T(K)	R	K_b_ (L mol^-1^)	n	ΔG (kJ mol^-1^)	ΔH (kJ mol^-1^)	ΔS (J mol^-1^·K^-1^)
288	0.9965	2.0 x 10^4^	0.9538	-23.73	-55.91	-111.74
300	0.9929	8.2 x 10^3^	0.8912	-22.39		
308	0.9933	4.3 x 10^3^	0.8501	-21.49		

#### Binding sites of LNF on the BSA

The binding sites for LNF on BSA were recognized with site marker competition experiments. Warfarin, ranitidine, phenylbutazone etc. are site I specific markers whereas ibuprofen, diazepam, flufenamic acid, etc. are site II specific markers of BSA [15, 37].

Warfarin and diazepam, are specific probes for site I and site II respectively. The competition experiment was carried out using different molar concentrations of LNF, whereas the BSA concentration and site specific probe concentration were equimolar and constant. Different concentrations of LNF were added to the BSA and the site probe mixture. The FI’s was measured at excitation intensity of 280 nm at room temperature. The change in the FI provides information about the binding site of LNF on bovine serum albumin. The obtained binding constant (Kb) at the room temperature in presence of specific binding probe were 1.4 × 10^3^ for the LNF and BSA with diazepam as probe and 5.3 × 10^3^ when warfarin was used as probe, whereas the LNF and BSA was found to be 8.2 × 10^3^. These values indicate that the binding constants decreased in presence of specific site probes and was the lowest in case of diazepam inferring competition of diazepam with ibuprofen for the same site. The experimental result indicates sub-domain IIIA of site II as the binding site on BSA molecule for LNF.

#### Interaction forces between LNF and BSA

There are several kinds of binding forces that might occur between the drug and the biomolecule. Thermodynamics viz. free energy change (ΔG^0^), enthalpy change (ΔH^0^), and entropy change (ΔS^0^) provide valuable information in understanding the interaction between the biomolecule and the drug. ΔH^0^>0 and ΔS^0^>0 indicate hydrophobic interaction, ΔH^0^< 0 and ΔS^0^< 0, reflect hydrogen bond formation or van der Waals force, whereas ΔH^0^ ≈ 0 and ΔS^0^> 0 suggest electrostatic force [38, 39]. To depict the intermolecular forces existing between LNF and BSA the temperature dependent thermodynamic system was utilized and evaluated at 288, 300 and 308 K. ΔH^0^ and ΔS^0^ for the interaction were determined using Van’t Hoff equation

$$\mathrm{lnKb}=-\frac{\Delta H^{0}}{RT}+\frac{\Delta S^{0}}{R}$$

$$\Delta G^{0}=\Delta H^{0}-T\Delta S^{0}=-RT\ln\;Kb$$

where Kb represent the binding constant at its corresponding temperature and R the gas constant. ΔG can be determined using van’t Hoff plot, where ΔH^0^ is the slope and ΔS the intercept.

Table 2 and Fig 2C contains the obtained values for ΔG^0^, ΔH^0^ and ΔS^0^. The spontaneous binding of drug to the biomolecule is represented by the negative sign of the ΔG^0^. The LNF and BSA interaction is mainly enthalpy driven is concluded from the negative (-) values of ΔS^0^ and ΔH^0^. The (-) value of the result suggests hydrogen bonding and/or van der Waals forces are present between LNF and BSA.

#### Ultraviolet–visible absorption study of BSA and LNF

UV-Visible spectroscopic studies provided information regarding both the structural changes if any and also complex formation. BSA absorption spectra alone and increasingly varied concentrations of LNF were recorded. It was observed that with increased LNF concentrations the absorption of BSA also showed an increase (blue shift of maximum peak of BSA at 280 nm) (Fig 1B). A possible justification for these two observations can be the creation of new BSA-LNF complex leading to change is protein conformation [40]

#### Synchronous fluorescence spectroscopic studies

The amino acids in BSA’s immediate vicinity is studied with synchronous fluorescence spectrum. The shift in the emission wavelength is linked with alteration in polarity of chromospheres [41]. Δλ = 15 and 60 nm represent the information of tyrosine and tryptophan on BSA molecule respectively. As it is obvious from that the FI decreases with increasing LNF concentration for both tyrosine and tryptophan residues. A slight shift of (1nm) was witnessed in the maximum wavelength when Δ λ = 15 nm whereas, no shift was witnessed when Δλ = 60 nm indicating slight alteration in tyrosine residues vicinity due to binding of LNF to BSA.

#### Transfer of energy from BSA to LNF

**Fig 3:**
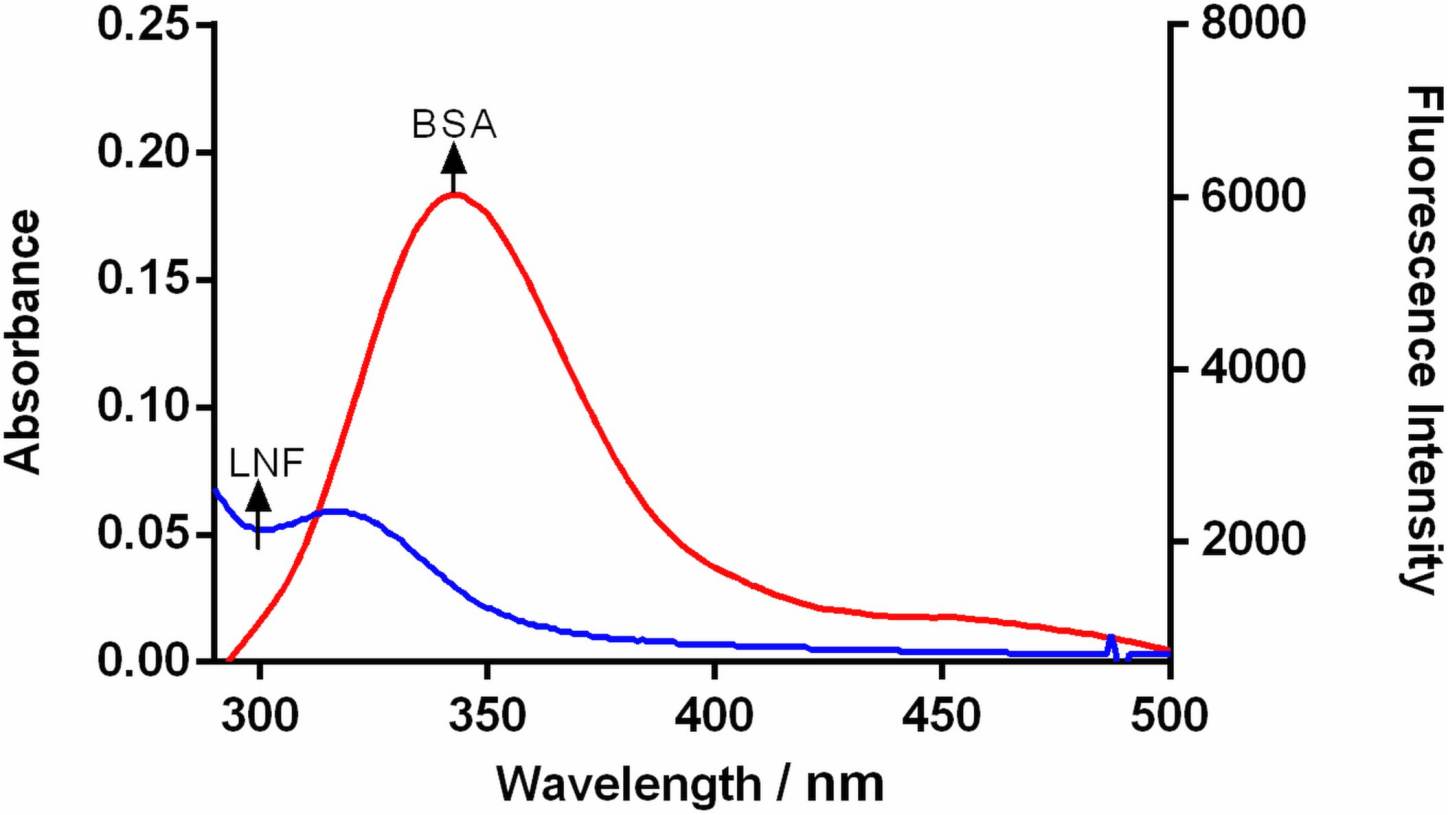
Fluorescence emission spectrum of BSA and UV-visible absorption spectra overlap.

Förster resonance energy transfer (FRET) technique is used to study biomolecule structure, distance of donor from acceptor, conformation, spatial distribution of complex proteins [42]. In order to calculate the FRET (E) among the LNF and BSA the complex formation should obey certain requirements: (1) there should be an overlap between the absorption of acceptor (Ligand) and fluorescence emission spectra of donor (BSA); The donor and acceptor are separated by a small distance of less than 8 nm[43]. This “E” amid the BSA and LNF (non fluorescent molecule) was estimated since the absorption of LNF (acceptor) overlapped significantly over the BSA emission spectra (donor) (Fig 3). The energy transfer (E) is represented as:

$$E=\frac{R_{0}^{6}}{\left(R_{0}^{6}+r^{6}\right)}=1-F/F_{0}$$

In the above equation the distance from donor to the acceptor is represented by r whereas R_0_ represents the distance of separation which will yield 50% of (E). F_0_ represents the FI of BSA without LNF and F represents the fluorescence intensity of BSA with LNF. R_0_ can be calculated by:

$$R_{0}^{6}=8.8\times 10^{-25}k^{2}n^{-4}\phi J$$

Where *k*^*2*^ is the spatial factor of orientation of the dipole and equals 2/3. ‘n’ represents the RI (refractive index) of the medium and equals 1.336. The quantum yield of fluorescence of the donor is denoted by *ϕ* and valued as 0.118 for BSA. The spectral overlap *J* (overlap between BSA and LNF) is calculated as.

$$J=\frac{\sum F\left(\lambda \right)\epsilon \left(\lambda \right)\lambda^{4}\Delta \lambda }{\sum F\left(\lambda \right)\Delta \lambda }$$

Where F(λ) denotes the donor FI at λ and ϵ(λ) denotes the extinction coefficient of acceptor at λ. From the equations above, the calculated values were, *J =* 5.43× 10^−14^, E = 0.12, R_0_ = 3.41 nm and r = 4.83 nm. From the results it is clear that the distance of the fluorophore residue of BSA and LNF is within the 2–8 nm range. Therefore, there is a high probability of energy transfer from BSA to LNF. Since, r > R_0,_ indicating static quenching.

### Molecular docking

To further understand the mechanism of interaction, molecular simulation studies were performed for BSA and LNF. The docking studies gave information regarding the binding sites available on BSA molecule in addition to binding energies of the ligand protein complex. The LNF (ligand) structure was drawn and the docking studies performed using Molecular Operating Environment-2014 (MOE-2014) software. The crystalline structure for BSA with PDB ID (4OR0) was acquired from Protein Data Bank (http://www.rcsb.org).

**Fig 4:**
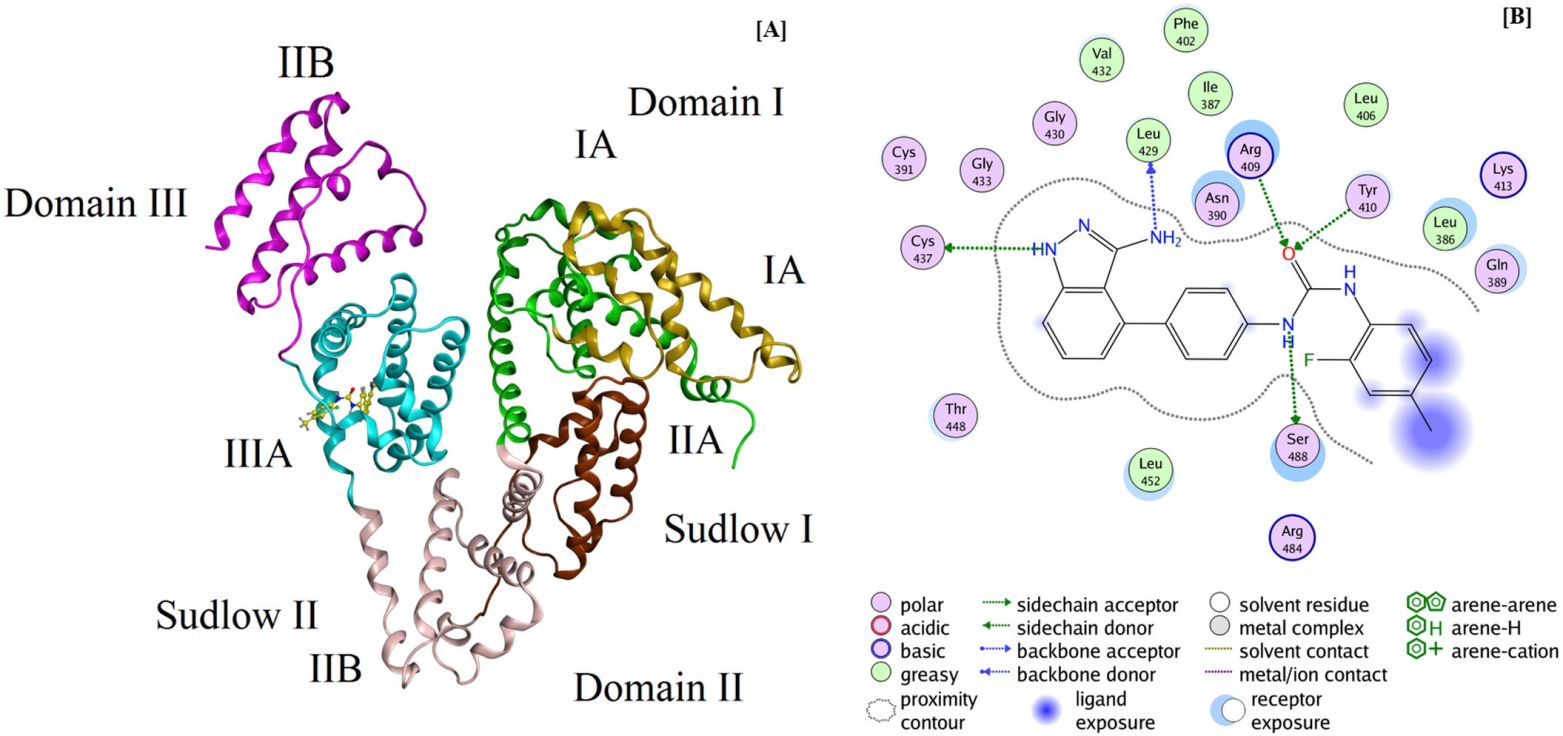
Molecular docking analysis results: [A] Best docking configuration for LNF sub-domain IIIA; [B] Amino acid residues surrounding bound LNF.

BSA protein has two binding sites namely site I (Sudlow’s sites I) and site II (Sudlow’s sites II). These binding sites are located at sub-domain IIA and IIIA of the hydrophobic regions. LNF binds to BSA on site II (sub-domain IIIA). Fig 4A provides the binding mode (best conformation) for interaction of LNF and BSA. The results from this study complement with the fluorescence and UV–vis spectroscopic studies and site marker experimental studies. The hydrogen bonding between LNF and amino acid residues and BSA molecule is depicted in Fig 4B and infer a very strong interaction between the two. Hydrogen bonds were seen between LNF and TYR-410, SER 488, CYS 437, LEU-429 and ARG-409 residues. This is in agreement with the experimental data where it was suggested hydrogen bonding and/or van der Waals forces are present between LNF and BSA. Moreover, five more amino acids GLN-389, ASN-390, LEU-386, LEU-452 and THR-448 encircle the LNF. The binding energy of the LNF–BSA complex from the docking studies was found to be -6.143 kcal/mol. The obtained value is slightly lower than the free energy obtained (-5.35 kcal mol^-1^) from the experimental data at 300K. These results further clarified the mechanism quenching of fluorescence of BSA by LNF. The molecular docking results are similar to those obtained from experimental studies carried out for determination of BSA-LNF interaction.

## Conclusions

The following conclusions were drawn from the experimental results

1. LNF binds to BSA to form a LNF-BSA complex was studied by UV–vis spectroscopy and fluorescence spectroscopy.
2. LNF binds to BSA resulting in a complex formation and static quenching of BSA intrinsic fluorescence. The LNF binds to BSA at sub-domain IIIA (site II).
3. The (-) ΔH^0^ and (-) ΔS^0^ during the thermodynamic studies indicate the role of hydrophobic forces in binding LNF and BSA.
4. UV–vis absorption and synchronous fluorescence confirmed that binding of LNF to BSA causes secondary structural variations of protein.
5. The study provides crucial facts regarding the LNF- BSA interaction and can prove to be of great pharmacological value for further investigations.
